# Effects of acetazolamide on linear growth in children with pseudotumor cerebri syndrome

**DOI:** 10.3389/fopht.2022.1042529

**Published:** 2023-02-06

**Authors:** Claire A. Sheldon, Sara N. Reggie, Grace L. Paley, Shana E. McCormack, Grant T. Liu

**Affiliations:** ^1^ Department of Ophthalmology, University of British Columbia, Vancouver, BC, Canada; ^2^ Ophthalmic Plastic & Cosmetic Surgery, Inc., St. Louis, MO, United States; ^3^ Department of Ophthalmology, Washington University, St. Louis, MO, United States; ^4^ Division of Endocrinology and Diabetes, Children’s Hospital of Philadelphia, Philadelphia, PA, United States; ^5^ Neuro-ophthalmology Service, Division of Ophthalmology, Children’s Hospital of Philadelphia, Philadelphia, PA, United States

**Keywords:** acetazolamide, pseudotumor cerebri (PTC), idiopathic intracranial hypertension (IIH), weight, growth

## Abstract

**Background/introduction:**

While oral acetazolamide is a cornerstone of management of adult and pediatric PTCS, previous studies have suggested that acetazolamide used in children with other conditions may influence growth.

**Aims and methods:**

Retrospective chart review involving a single tertiary medical center. Thirty-four children with definite or probable PTCS were identified. Analysis was restricted to individuals from whom anthropometric data were available *before* and *during* acetazolamide treatment (n=22).

**Results:**

Half of individuals (n=11/22) showed a decline in BMI Z-scores. Sixty-three percent (n=14/22) showed a decrease in height Z-scores during treatment with acetazolamide; in 6 of these 14 children who had complete data, 3 showed at least a partial recovery of height Z-scores after discontinuation of acetazolamide.

**Conclusion:**

Acetazolamide may be associated with growth suppression in some children treated for pediatric PTCS. In some cases, the growth suppression appears to reverse once the acetazolamide is stopped.

## Introduction

Pseudotumor cerebri syndrome (PTCS) encompasses the constellation of symptoms caused by elevated intracranial pressure of unclear etiology with normal brain parenchyma and cerebrospinal fluid constituents (1). Pediatric PTCS shares some, but not all, features of its adult counterpart. For example, previous studies suggested age, sex, and pubertal status influence the epidemiology of pediatric PTCS (2–6). Both female sex and obesity appear to be more strongly associated with PTCS in older, but not younger, pediatric patients (2, 7). In addition, further analysis has characterized subgroups of pediatric PTCS: a ‘young’ cohort, with normal height and weight, an ‘early adolescent’ cohort who are typically overweight, and a ‘late adolescent’ cohort who are typically obese (8).

While oral acetazolamide is a cornerstone of management of adult and pediatric PTCS, previous studies have suggested that acetazolamide used in children with other conditions may influence weight gain and growth (9, 10). One study retrospectively reviewed medical records of children (n=22, age range 2 months to 15 years) on acetazolamide for management of glaucoma. Nine percent showed a decline in weight gain which crossed at least two growth percentile categories (e.g., from 50^th^ percentile to <10^th^ percentile) during acetazolamide treatment (9). In a second study (n=17 subjects, age range 1.2 – 6.5 years), the use of acetazolamide, as an adjunct management of epilepsy, was associated with a significant decrease in both height and weight standardized scores (10).

While adults with PTCS on acetazolamide do not have to be concerned about growth suppression, this potential side effect would be disconcerting to growing children with PTCS and their parents. Our study examines the effects of oral acetazolamide on pediatric growth parameters during treatment for PTCS.

## Methods

This study is a retrospective chart review involving a single tertiary medical center. Institutional Review Board (IRB)/Ethics Committee approval was obtained (Children’s Hospital of Philadelphia (IRB# 13-010158). Patient charts were identified *via* an electronic medical record search for ICD-9 code 348.2 and/or patient database of a single pediatric neuro-ophthalmologist (GTL). For this pediatric study, only patients aged 2 to 18 years at diagnosis were included in the study, as we considered the pathophysiology of this diagnosis in infancy to be distinct. Cases of definite or probable PTCS were collected, as previously described (1, 8). Cases of primary PTCS (also known as idiopathic intracranial hypertension, IIH) and secondary PTCS were included. Retrospective data were collected on patients seen between July 1993 and April 2013 as part of a previous study (8), using manual and bioinformatics-based abstraction. During chart review, all available measurements of height and weight were collected, at time points prior to and following the diagnosis of PTCS until April 2013.

Analysis was limited to subjects with ≥ 5 growth parameter measurements over time and clear documentation of acetazolamide use, with standard oral dosing of 15-20 mg/kg/day divided into two or three doses. For each case, age and anthropometric measurements were documented according to U.S. CDC 2000 growth standards (11). Anthropometric measurements included height Z-scores, weight Z-scores, and body mass index (BMI) Z-scores. BMI Z-scores were categorized as obtained prior to, during, and after discontinuation of acetazolamide. Where more than one measurement was available, mean data was reported and included for analysis. As in previous studies, pediatric obesity was defined using BMI Z-score. Overweight and obese were defined according to the CDC classifications of overweight (BMI percentile for age and sex ≥ 85 and < 95, corresponding to BMI Z-score ≥ 1.04 and < 1.64) and obese (BMI percentile for age and sex ≥ 95, corresponding to BMI Z-score ≥ 1.64) in children. Study data were collected and managed using REDCap (Research Electronic Data Capture) tools (12) hosted at The Children’s Hospital of Philadelphia.

## Results

**Figure 1:**
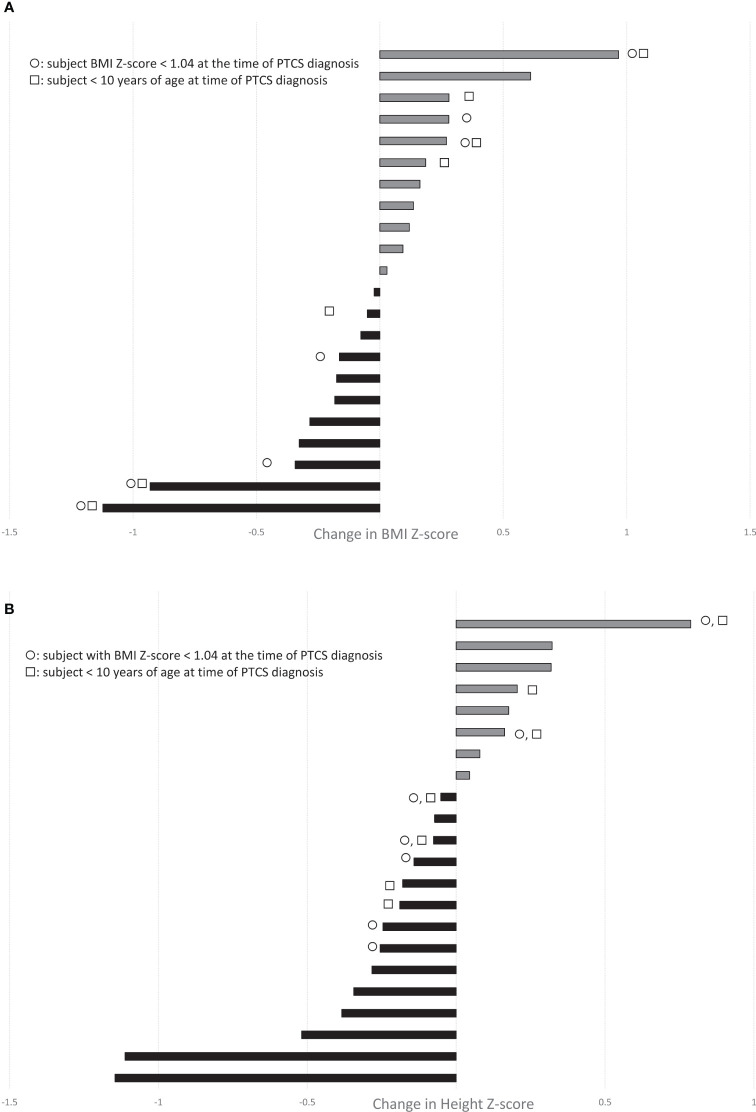
Waterfall plots demonstrate the change in **(A)** BMI-Z score and **(B)** Height Z-score during treatment with acetazolamide in our cohort of children with PTCS (range, 1-32 months; n=22 with paired growth data available before and during treatment). Grey bars depict increases, while black bars show decreases. ◯ indicates subjects with BMI Z-score < 1.04 (not overweight or obese) at the time of PTCS diagnosis. □ indicates subjects < 10 yrs of age at PTCS diagnosis.

Using updated PTCS diagnostic criteria (1), we identified 34 pediatric subjects with definite or probable PTCS treated with acetazolamide, of whom 29 were diagnosed with primary PTCS. The other 5 were diagnosed with secondary PTCS (three had renal failure, one used doxycycline, and the other was withdrawn from chronic corticosteroids). Subjects were 59% female with a mean age at diagnosis of 12.5 ± 4.1 years and mean BMI Z-score at diagnosis of 1.16 ± 1.1 (mean ± standard deviation, **Table 1**). The duration of acetazolamide treatment ranged from 49 – 982 days, with a median of 201 days. For subjects with anthropometric data available *before* and *during* acetazolamide treatment (n=22), there was no obvious trend in change in BMI Z-score as half subjects showed a decline in BMI Z-scores (n=11/22), while the other half had an increase in BMI Z-score (**Figure 1A**). In addition, 63% of subjects showed a decrease in height Z-scores during treatment of acetazolamide (n = 14/22) (**Figure 1B**).

**Table 1:** Initial demographics and anthropometrics of pediatric subjects diagnosed with PTCS and included in the evaluation of the effect of acetazolamide on growth parameters.

Characteristic	
Sex (% female, n)	59% (20/34)
Age (yrs)	12.5 ± 4.1
Height	
Height (m)	1.49 ± 0.21
Height Z-score	0.46 ± 1.22
Weight	
Weight (kg)	62.8 ± 29.2
Weight Z-score	1.34 ± 0.97
BMI	
BMI (kg/m^2^)	25.3 ± 8.4
BMI Z-score	1.16 ± 1.10

Summary outcomes for continuous variables are presented in means ± standard deviation.

We questioned whether that the effects of acetazolamide on weight gain and growth might depend on baseline nutritional status or age, but some children who were of normal weight or less (ie. BMI Z-scores <1.04) and/or young (age <10) experienced a decrease in height Z-score, while there were others who had an increase in height Z-score (**Figure 1B**).

**Figure 2:**
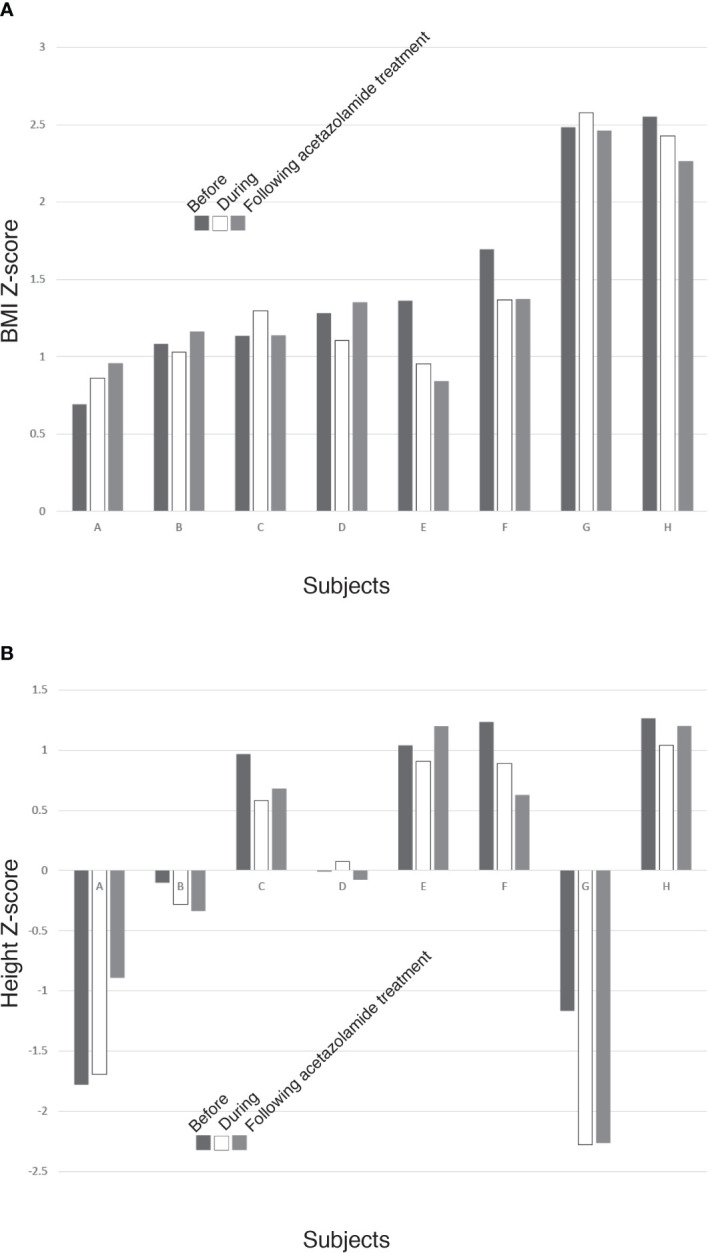
Bar graphs showing **(A)** BMI Z-scores & **(B)** Height Z-scores measured before (black bar) during (white bar) and following (grey bar) treatment with acetazolamide in the 8 subjects (A–H) who had those three measurements. In **(A)** 5 children (B, D, E, F, H) had a decrease in BMI Z-scores during treatment with acetazolamide. In **(B)**, 6 children (B, C, E, F, G, H) developed a decrease in height Z-scores during treatment, and of these, 3 (C, E, H) showed at least a partial recovery of height Z-scores after discontinuation of acetazolamide.

Limited paired data were available for children before, during and after discontinuation of treatment (*n*=8, with > 1 measurement of anthropometric before, during, after discontinuation treatment with acetazolamide; follow-up anthropometric data was obtained after discontinuing acetazolamide over a range of 6 – 33 months). Qualitative analysis suggests the possible effects of acetazolamide on growth suppression may, in part, be reversible. Six children developed a decrease in height Z-scores during treatment with acetazolamide, and of these, 3 showed at least a partial recovery of height Z-scores after discontinuation of the drug (**Figure 2A**). Five had a decrease in BMI Z-scores during treatment with acetazolamide (**Figure 2B**).

## Discussion & conclusion

Previous studies on other diseases have suggested that in some susceptible children with other diseases, acetazolamide can suppress growth. If acetazolamide plays a causal role in growth suppression in some children with PTCS, the mechanism is not entirely clear. To our knowledge, the effect of acetazolamide on growth in pediatric PTCS has not been evaluated systematically. We found that some children with PTCS developed growth suppression during acetazolamide treatment. There was no obvious relationship to young age or weight. Fortunately, the possible effect of acetazolamide on growth may be reversible after treatment cessation, at least for the treatment durations captured in our review. Future larger prospective studies are required to confirm our findings.

How acetazolamide may lead to growth suppression in this group remains unclear. Prior studies have proposed metabolic acidosis induced by acetazolamide as the mechanism by which growth retardation is induced in children; however, downstream details are unclear, with varied hypotheses including alterations in thyroid hormone homeostasis and blunting of growth hormone secretion (10). *In vitro* studies have illustrated that acetazolamide may decrease lipogenesis in adipose cells, as well as potentiate growth inhibition and induce cell cycle arrest and apoptosis in other model systems (13). Clinically, a handful of studies have demonstrated the use of acetazolamide for weight loss in adults without PTCS. For example, acetazolamide has been used for the management of antipsychotic-associated weight gain, in a manner analogous to the effects seen with topiramate (14, 15). Whether these effects occur in the pediatric population and, the extent to which they depend on the dose and/or duration of acetazolamide therapy is unknown.

The retrospective nature and small sample size are the largest limitations of this study. The majority of patients are scheduled at regular intervals based on practitioner practice (GTL); however, visits to the neuro-ophthalmologist naturally do vary, inducing possible sampling bias for measurement of height and weight. Further prospective studies are needed, with additional recording of dosing and compliance with acetazolamide treatment.

Despite these limitations, our findings suggest that acetazolamide may be associated with growth suppression in some children during the management of pediatric PTCS. Fortunately, in several cases, the growth suppression appears to reverse at least in part once the acetazolamide is stopped. Because of the findings of this study, 1) before starting acetazolamide in children we discuss with parents this potential side effect but also its reversibility, and 2) when papilledema is improving, we might halve the dose of acetazolamide or discontinue it if growth suppression occurs. This study emphasizes the necessity to measure routinely the height and weight of children during and after discontinuation of acetazolamide in the management of pediatric PTCS, and also underscores the need for further prospective study into this potentially deleterious effect of acetazolamide. We are currently designing an multicenter prospective pediatric PTCS study to characterize better the clinical and laboratory features of PTCS in this age group as well as to further our understanding of the disease mechanism in children. In this study we will include an analysis of the side effects of acetazolamide in children with PTCS.

## Data availability statement

The original contributions presented in the study are included in the article. Further inquiries can be directed to the corresponding author.

## Ethics statement

The studies involving human participants were reviewed and approved by Children’s Hospital of Philadelphia Institutional Review Board (IRB)/Ethics Committee. Written informed consent from the participants’ legal guardian/next of kin was not required to participate in this study in accordance with the institutional requirements.

## Author contributions

All authors listed have made a substantial, direct, and intellectual contribution to the work, and approved it for publication.
